# Supplementary material for: Isolation of Reporter Cells That Respond to Vitamin A and/or D Using a piggyBac Transposon Promoter-Trapping Vector System
Source: Int J Mol Sci. 2022 Aug 19;23(16):9366. doi: 10.3390/ijms23169366 (PMC9409033; doi:10.3390/ijms23169366)
Supplement: Supplementary file 1 [file ijms-23-09366-s001.zip › Supplement(FigS1&S2).pdf]

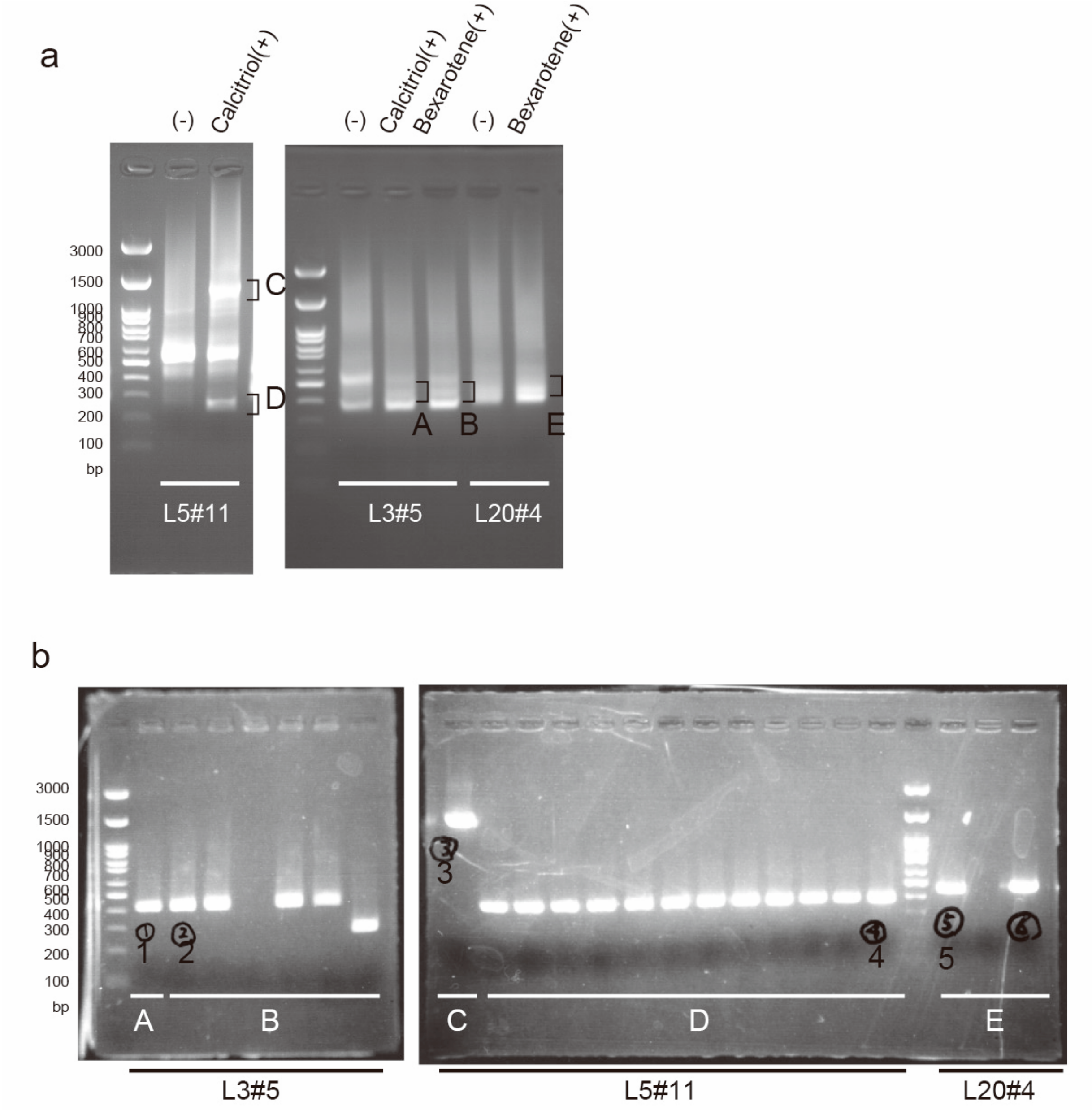

Figure S1. (**a**) 5′ RACE to identify responsive genes fused to *P2A-GAL4FF* and (**b**) colony PCR analysis for subsequent cloning of the 5′ RACE cDNA fragment (A–E in (a)) into pBluescript II. They were performed exactly as described previously [4].

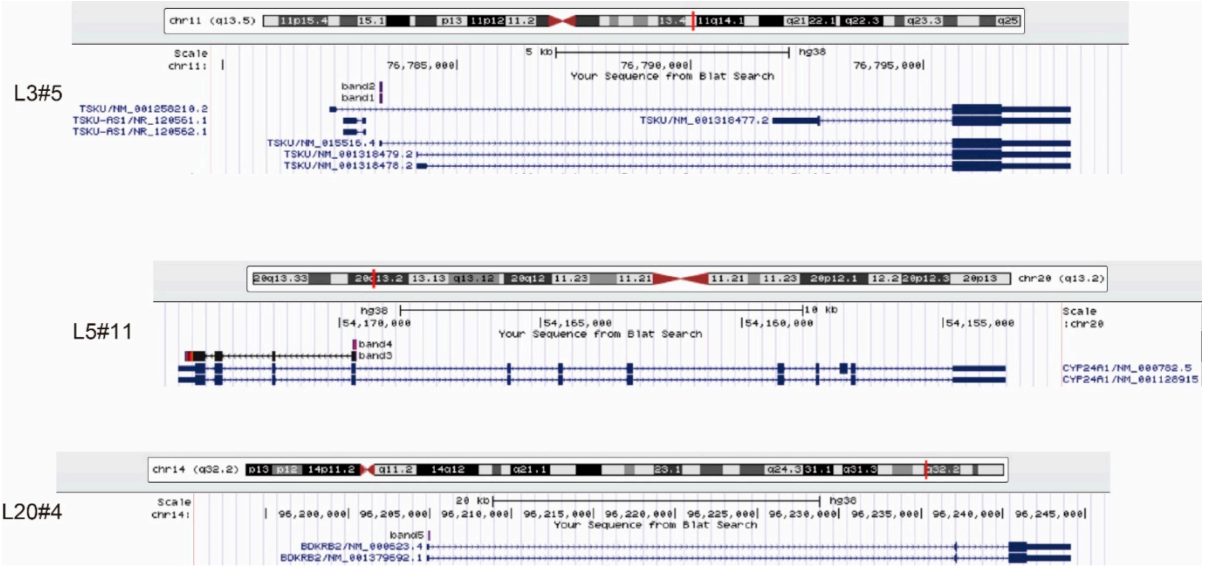

Figure S2. Mapping of the DNA fragment cloned using 5′ RACE for genes fused to *P2A-GAL4FF*. The DNA fragment shown in Figure S1(b) was collected, purified, read by a reverse primer (Supplementary Data S1–5), and analyzed using the BLAT tool (https://genome.ucsc.edu/cgi-bin/hgBlat).
